# Serum Amyloid A Correlates With the Osteonecrosis of Femoral Head by Affecting Bone Metabolism

**DOI:** 10.3389/fphar.2021.767243

**Published:** 2021-10-18

**Authors:** Xiaoyuan Peng, Yiyang Ma, Qiyang Wang, Yanchun Gao, Guangyi Li, Chenyi Jiang, Yun Gao, Yong Feng

**Affiliations:** Department of Orthopedic Surgery, Shanghai Jiao Tong University Affiliated Sixth People’s Hospital, Shanghai, China

**Keywords:** serum amyloid a, osteonecrosis of femoral head, proteomics, wnt/β-catenin signaling pathway, MAPK/PPARγ signaling pathway

## Abstract

Osteonecrosis of femoral head (ONFH) is a progressive hip joint disease without disease-modifying treatment. Lacking understanding of the pathophysiological process of ONFH has become the humper to develop therapeutic approach. Serum amyloid A (SAA) is an acute phase lipophilic protein during inflammation and we found that SAA is increased for the first time in the serum of ONFH patients through proteomic studies and quantitatively verified by ELISA. Treating rBMSCs with SAA inhibited the osteogenic differentiation *via* Wnt/β-catenin signaling pathway deactivation and enhanced the adipogenic differentiation *via* MAPK/PPARγ signaling pathway activation. Finally, bilateral critical-sized calvarial-defect rat model which received SAA treated rBMSCs demonstrated reduction of bone formation when compared to untreated rBMSCs implantation control. Hence, SAA is a vital protein in the physiological process of ONFH and can act as a potential therapeutic target to treat ONFH.

## Introduction

Osteonecrosis of femoral head (ONFH) is a progressive hip joint disorder without consensus of effective treatment, ultimately leading to total hip replacement (Mont et al., 2006). The pathogenesis of ONFH is perplexing and needs further investigation (Hines et al., 2021). Use of steroid, overuse of alcohol and trauma of femoral neck or head are the three well known risk factors for ONFH. Previous study has indicated that the pathogenesis of ONFH is related to the severe degradation of the bone tissue (Weinstein et al., 2000), promoted differentiation of bone mesenchymal stem cells (BMSCs) to adipocytes and hypertrophy of the adipocyte through increasing intracellular lipid synthesis (Peckett et al., 2011). The increase of marrow fat cell induces intra-osseous hypertension in the proximal femur. Venous sinusoids are compressed due to the intra-osseous hypertension and then intravascular coagulation occurs. Arterial blood flow is blocked and eventually results in ischemia in the femoral head (Yoon et al., 2020). However, the specific underlying molecular mechanism of aberrant bone metabolic balance has not been completely elucidated.

Serum amyloid A (SAA) is a family of acute phase response proteins which is coded by various genes, which exhibit high degrees of allelic variation and mammalian homology (Uhlar et al., 1994; Lloyd-Price et al., 2019). Apart from high levels of SAA found in the liver (Strnad et al., 2017), the protein has been found to be expressed in monocytes/macrophages (Meek et al., 1992), chondrocytes (Zerega et al., 2004) and adipocytes (Han et al., 2020). Meanwhile, SAA is thought as a crucial marker of inflammation and a precursor protein of amyloidosis involved in cellular cholesterol homeostasis, promotion of signalling cascades, and modulation of intercellular calcium levels (Artl et al., 2000; Baranova et al., 2005; Husebekk et al., 2010). Previous study has indicated that SAA is a lipid soluble protein, of which the 95% surface has lipophilic receptors, especially for high density lipoprotein (HDL) (Frame and Gursky, 2017). In particular, SAA is involved with cholesterol metabolism in both physiologic and inflammatory conditions (Sack, 2018). Clearly, the intimate relationship among SAA/HDL/cholesterol has implicated SAA in the pathogenesis of various diseases, such as rheumatoid arthritis (Mun et al., 2021), atherosclerosis (Webb, 2021), alcoholic liver cirrhosis (Kim et al., 2014) and insulin resistance (Li et al., 2021). Mechanistically, SAA participates in the processes of the above diseases by binding with HDL, promoting adipogenic differentiation and promoting the proliferation of preadipocytes. In this study, we aimed to explore the role of SAA in bone metabolism to deepen the research on the pathophysiologic mechanism of ONFH.

In an effort to better understand those mechanisms underlying ONFH, proteomics has emerged as a well-defined strategy which, through in-depth characterization of the whole tissues, sera, and biofluid proteome, has provided substantial data in terms of potential protein biomarkers associated with the mechanism pathways and prognosis of disease (Pan et al., 2005; Veenstra et al., 2005; Zapico Muñiz et al., 2005). In this study, the serum proteome technology was exploited to assess the differentiate expression levels of serum proteins between ONFH patients and healthy controls. Based on the results of proteomics, SAA was hypothesised to exhibit a correlation with ONFH, and this assumption was confirmed with ELISA. The influence of SAA on the osteogenic and adipogenic differentiation of BMSCs was determined to confirm the link between SAA and ONFH. Combined with the results of *in vitro* experiments, the effect of SAA on bone metabolism had also been verified *in vivo* experiments. This work will provide us with the in-depth knowledge of SAA-induced bone metabolism disorder and ONFH pathogenesis.

## Material and Methods

### Proteomics Analysis

#### Patients

For proteome analysis, the steroid-induced ONFH group comprised 11 patients who received corticosteroid medication. These patients were diagnosed with ONFH from magnetic resonance imaging (MRI) findings and were not treated for ONFH. A set of 11 healthy volunteers matched with sex and age was included as the control group. The clinical features and demographic information of the steroid-induced ONFH group and the control group were summarized in Supplementary Material. For ELISA, 20 patients with steroid-induced ONFH, 20 with alcohol-induced ONFH, 20 with trauma-induced ONFH, and 20 healthy volunteers were enrolled. All the patients and volunteers participating in the ELISA study did not have underlying factors other than ONFH that might cause the elevation of SAA. The clinical features and demographic information of the patients and volunteers were summarized in Supplementary Material. All procedures performed in this study involving human participants were in accordance with the ethical standards of the institutional and/or national research committee and with the 1964 Declaration of Helsinki and its later amendments or comparable ethical standards. Informed consent was obtained from all individual participants included in the study.

### Preparation of Serum Samples

A total of 5 ml of peripheral venous blood was withdrawn from patients and healthy volunteers. The blood samples were coagulated at 37°C for 15 min and centrifuged at 2,000 × g for 15 min. The supernatant was collected and stored at −80°C.

#### Elimination of Highly Abundant Proteins and Quantification

The serum samples were thawed and attenuated at 37°C. The attenuated serum samples were percolated with filter membranes (0.22 μm pore size). The 14 most abundant proteins were depleted from the plasma using the multiple affinity removal column system (MARS, Agilent Technologies spin columns, United States) according to the manufacturer’s protocol. The depleted sample was buffer exchanged with 50 mm ammonium bicarbonate using Vivaspin concentrator (5,000 molecular weight cut-off, Sartorius Group, Germany). The proteins from the processed serum samples were quantified using a protein assay reagent kit (Bio-Rad Laboratories Inc, Hercules, CA, United States) and stored at −80°C.

#### Two-Dimensional Gel Electrophoresis

The serum samples were subjected to two-dimensional gel electrophoresis. Briefly, 100 μg of each serum sample was loaded onto an immobilized pH gradient (IPG) strip (GE Amersham, United Kingdom) with pH variation from 3 to 10. The first dimensional isoelectric focusing was implemented as follows: 30 V for 12 h, 500 V for 1 h, 1,000 V for 1 h, 8,000 V for 8 h, and 500 V for 4 h using Ettan IPGphor Isoelectric Focusing System (GE Amersham, United Kingdom). The proteins after iso-electrophoresis were separated with second dimensional sodium dodecyl sulphate polyacrylamide gel electrophoresis (SDS-PAGE) using Hofer SE 600 (GE Amersham, United Kingdom). The second-dimension gel electrophoresis was performed on the 12.5% SDS-polyacrylamide slab gels at 15 mA for 30 min and 30 mA until the trace of bromophenol blue was 0.5 cm away from the bottom of the gel.

#### Silver Staining

The silver staining of the gels after electrophoresis was performed according to the modified silver-staining protocol with the Silver Stain PlusOne kit (GE Amersham, United Kingdom). The gels were fixed with 50% methanol and 5% ethyl alcohol for 1 h, sensitized with sensitizing solution comprising 0.2% sodium thiosulphate, 30% methanol, and 68 g/L sodium acetate for 5 min, washed thrice with distilled water for 5 min, incubated with 0.25% silver nitrate for 20 min, and rinsed thrice with distilled water for 1 min. The gels were developed with 0.04% formaldehyde and 2% sodium carbonate by vigorous shaking and the reaction was terminated with 5% acetic acid till the coloration was moderate.

#### Gel Imaging

The silver-stained gels were scanned with UMax Powerlook 2110XL (GE Amersham, United Kingdom) and the spot patterns of the gel images were matched using Image Master 2D system (Amersham Biosciences). The densities of all the matched spots were standardized with the total protein amount in the gel.

#### Destaining and Trypsin Digestion

Different groups of spots were excised from the gel and rinsed with de-ionised water. The washed gommures were destained with 50 μl of a solution containing 30 mmol/L potassium ferricyanide (K_3_Fe(CN)_6_) and 100 mmol/L of sodium thiosulfate (Na_2_S_2_O_3_) at a ratio of 1:1. After lyophilization, digestion was performed with 5 μL of trypsin (Promega, United States) at 37°C for 20 h. The enzymatic hydrolysate was subsequently extracted, and the residual was treated with 100 μl of 60% acetonitrile (ACN) and 0.1% trifluoroacetic acid (TFA). The samples were ultrasonically washed for 15 min and the cleaned solution obtained was merged with the enzymatic hydrolysate. The collected fluid was desalted with ZipTip (Millipore, United States) and freeze dried.

#### Matrix-Assisted Laser Desorption Ionisation Time-Of-Flight Mass Spectrometry

The dried samples were dissolved in 2 μl of 20% acetonitrile and treated with supersaturated *a*-cyano-4-hydroxycinnamic acid (CHCA) matrix solution. The solvent composed of 0.5% TFA and 50% ACN. All the samples were analyzed on 4800 Plus MALDI TOF/TOFTM Analyzer (Applied Biosystems, United States) at a scan range of 800–4,000 Da with UV light at 355 nm using Nd:YAG laser. The data were searched using the International Protein Index human protein database with Mascot search engine.

#### ELISA

The serum levels of the identified protein, SAA, were determined with ELISA performed using the human SAA ELISA Kit (Anogen, Canada) according to the manufacturer’s instructions. The patients and volunteers were enrolled in the study and the serum samples were obtained as mentioned before.

### Vitro Experiment

#### Isolation and Cultivation of rBMSCs

RBMSCs were obtained from Sprague-Dawley male rats. Briefly, the marrow from the femur and tibia of male rats was flushed under aseptic conditions. For the isolation of rBMSCs, the cell suspension was sifted with a 400-mesh filter. The filtered cell suspension was centrifuged for 5 min at 4°C. The floating fraction was discarded, and the enriched cells were treated with an erythrocyte lysis buffer. rBMSCs were suspended and transferred into culture flasks and cultured with *α*-Modified Eagle Medium (*α*-MEM, HyClone, United States), supplemented with 10% foetal bovine serum (FBS, Gibco, United States) and 1/100 penicillin-streptomycin (Gibco). The cells were maintained in a humidified atmosphere containing 5% CO_2_ at 37°C, and the culture medium was replaced every 2 days. After three to five passages, rBMSCs were used for subsequent experiments. For osteogenic differentiation, rBMSCs were incubated in culture medium supplemented with 10^–2^ M *ß*-sodium glycerophosphate, 50 μg/ml of l-ascorbic acid, and 10^–7^ M dexamethasone for 14 days. For adipogenic differentiation, the cells were incubated with an adipogenic induction culture medium (Cyagen, China) for 2 wk.

#### Cell Counting Kit-8 (CCK-8) Assay

Direct cell counting was performed to compare the proliferative rate of rBMSCs untreated or treated with SAA (PeproTech, United States). A total of 1 × 10^–4^ rBMSCs were seeded in a 96-well plate and treated as the group information mentioned before. The toxicity and proliferation of cells were detected with CCK-8 kit (Beyotime, China). At each time point (Day 1, 3, and 7), the proliferative rate of rBMSCs was assessed according to the manufacturer’s instructions.

#### Bromo-4-chloro-3-indolyl-phosphate (BCIP)/Nitro Blue Tetrazolium (NBT) Staining

To visualise the alkaline phosphatase (ALP) activity, the BCIP/NBT staining was used. rBMSCs were seeded into a 24-well plate at 1 × 10^–5^ cells per well. Different groups of rBMSCs were cultured in osteogenic induction culture medium for 7 days. The culture medium was renewed every 2 days. Following treatment, the cells were washed with phosphate-buffered saline (PBS) and fixed with 4% paraformaldehyde for 20 min at room temperature. The fixed cells were re-washed thrice with PBS and treated with BCIP/NBT solution (Beyotime, China) in the dark for 1 h at room temperature. Images were observed and acquired using a microscope (Leica, Germany) and digital camera (Canon, Japan). The ALP activity in the cellular fraction was measured using a microplate test kit (Nanjing Jiancheng Biotechnology Co Ltd Jiangsu, China) following the manufacturer’s instructions, and the absorbance at 520 nm was measured using a microplate reader.

#### Alizarin Red Staining

Alizarin red staining is widely used to evaluate the osteogenic differentiation of cells based on the detection of calcium deposition in the extracellular matrix that serves as a marker of early osteogenesis. After co-cultivation with osteogenic induction medium and SAA for 14 days, different groups of rBMSCs were fixed with 4% paraformaldehyde for 20 min and washed thrice with PBS at 37°C. The cells were stained with alizarin red solution (Cyagen, China) for 30 min, and images of general view were captured with digital camera under 10× using microscope. To quantify mineralization, the calcium deposition was desorbed with 10% cetylpyridinium chloride (Sigma-Aldrich), after which the solution was collected, and the OD was measured at 570 nm.

#### Oil Red O Staining

Oil red O staining was performed after 14 days of treatment with adipogenic induction culture medium (Cyagen, China) to evaluate the adipogenic differentiation of various groups of rBMSCs. The cells were fixed with 4% paraformaldehyde for 20 min, rinsed thrice with PBS, and stained with Oil red O solution comprising PBS and 0.5% oil red O stock solution. The neutral lipids were stained red and served as the biomarker of adipogenesis. Microscope was used to capture images of the stained cells under 10× magnification. After the observation, isopropanol was used to release the stain, and the degree of adipogenesis was detected by measuring at 540 nm.

#### Western Blot Analysis

The rBMSCs were plated in a six-well plate and cultured until they reached 90% confluency. The cells were induced with osteogenesis or adipogenesis culture medium and incubated in the presence of 150 mg/L of SAA for 7 days. The proteins were subsequently collected after treatment with the combination of cell lysis buffer, phosphatase inhibitor, proteinase inhibitor, their concentration was determined with bicinchoninic acid protein assay kit (Cell Signalling Technology, Shanghai, China). Equal amounts of proteins (20 μg) were separated on SDS-PAGE gels by electrophoresis and transferred onto polyvinylidene difluoride (PVDF) membranes. The membranes were blocked with a blocking buffer for 1 h and incubated with the corresponding primary antibody at room temperature. After washing thrice with TBST, the membranes were incubated with horseradish peroxidase (HRP)-conjugated polyclonal goat antibodies, *ß*-actin or glyceraldehyde 3-phosphate dehydrogenase (GAPDH) were used as internal references. Extracellular signal-regulated kinase (ERK)-1/2, phospho-ERK1/2, glycogen synthase kinase 3 beta (GSK3β) and phospho-GSK3βantibodies were purchased from Cell Signalling Technology (Shanghai, China), while *ß*-catenin antibodies were obtained from Abcam (United Kingdom). Peroxisome proliferator-activated receptor gamma (PPARγ) and GAPDH antibodies were supplied by Servicebio (Wuhan, China).

#### Real-Time Reverse-Transcription Polymerase Chain Reaction (RT-PCR)

The mRNA expression of osteogenic differentiation-related genes (*ALP*, *OCN*, *Runx2*, and *COL-1*) and adipogenic differentiation-related genes (*aP2*, *Adipoq*, and *PPARγ*) was assessed with real-time RT-PCR. After 7 days of incubation in an appropriate induction medium and stimulation with various concentrations of SAA, the total RNA was extracted from cells using EZ-press RNA purification Kit (EZ Bioscience, China) and reverse transcribed to generate complementary DNA using 4× Reverse Transcription Master Mix (EZ Bioscience, China). The forward and reverse primers (BioTNT, China) for cDNAs were designed as indicated in Table 1. A total of 10 μl of mixture comprising 1 μl of cDNA, 0.3 μl of forward primer, 0.3 μl of reverse primer, 5 μl of qPCR SuperMix (BioTNT, China), and 3.4 μl of distillation-distillation H_2_O was loaded to each well of a 384-well plate and real-time RT-PCR was performed on 7900HT Fast Real-Time PCR System (Thermo Fisher Scientific, United States). The thermal cycle for RT-PCR was as followed: 95°C for 30 s; 40 cycles at 95°C for 10 s and at 60°C for 30 s. The expression of mRNAs was calculated by the 2^−△△Ct^ method and the expression of target gene was normalised to that of *ß*-actin.

**TABLE 1:** List of forward and reverse primers used for RT-PCR.

Primer	Forward	Reverse
Alp	5′-CGT TGA CTG TGG TTA CTG CTG-3′	5′-CTT CTT GTC CGT GTC GCT-3′
OCN	5′-CAG ACA AGT CCC ACA CAG CA-3′	5′-CCA GCA GAG TGA GCA GAG AGA-3′
COL-1	5′-TGT GCG ATG GCG TGC TAT-3′	5′-CCT ATG ACT TCT GCG TCT GGT G-3′
Runx2	5′-ATC ATT CAG TGA CAC CAC CAG-3′	5′-GTA GGG GCT AAA GGC AAA AG-3′
PPARγ	5′-CCT CTC TGT GAT GGA TGA CCA-3′	5′-ACA TCC CGT TCA CAA GAG CT-3′
aP2	5′-TGA AAC TGA CGA TCA CAC AGG-3′	5′-ACA GAA CTC ACT GGG ACC TGG-3′
Adipoq	5′-ATG ATA CCA ACT GAC TGC CAC T-3′	5′-TTG CTT ACT TTG AGG GTT CTG A-3′
*β*-actin	5′-CCT CTA TGC CAA CAC AGT-3′	5′-AGC CAC CAA TCC ACA CAG-3′

### Vivo Experiment

#### Bilateral Critical-Sized Calvarial-Defect Model

To test the ability of SAA affecting bone metabolism *in vivo*, fifteen 12-wk-old male SpragueeDawley rats (body weight: 250–300 g) were obtained from the experimental animal centre at the Hospital, China. Prior to surgery, rats were anesthetized by pentobarbital sodium through intraperitoneal injection. The rats’ heads were stabilized with a stereotactic frame to prevent movement during surgical procedures. The surgical areas were shaved, and the skin was disinfected with 75% ethanol. A 1.5–2 cm length mid-sagittal skin incision was created on scalp, and two 5°mm-diameter, critical size, circular bone defects were created with electric trephine drill (Nouvag AG; Goldach, Switzerland) with a low-speed handpiece under continuous saline irrigation to both parietal bones bilaterally symmetrical by the reference of the bregma point and sagittal suture. ShakeGel™ 3D hydrogel (Biomaterials United States, VA, United States) was used as the scaffold to load rBMSCs. Cells were mixed into the hydrogel following the manufacturer’s protocol, and the cell/hydrogel composites were then applied to the calvarial bone defects in rats. For groups implanted with rBMSCs treated with SAA, the cells were precultured with 150 mg/L SAA for 5 days before being mixed with the hydrogel. To ensure that the cells were exposed to SAA *in vivo*, SAA was added to the mixed hydrogel composites at a final concentration of 150 mg/L. All the animals were permitted access to food and water freely and daily observe for potential complications or abnormal behaviour. All experimental procedures were approved by the Animal Research Committee of the Hospital.

#### Micro-CT Scanning Analysis

Twelve-weeks after bilateral critical-sized calvarial-defect model establishment, all the rats were euthanized. The craniums of rats were scanned by a microCT scanner (Bruker, Germany) and the 2-D images were analysed by CTAn software (Bruker, Germany). For the craniums, the parameters of the new bone volume/total volume (BV/TV), bone mineral density (BMD) and Trabecular thickness (TbTh) of the bone defect area were recorded and collected for analysis.

#### Immunofluorescence Staining

Polychrome sequential fluorescent labelling was conducted to observe the rate of new bone formation and mineralization. The rats were intraperitoneally injected fluorochromes under anaesthesia as follow, 25 mg/kg tetracycline, (Sigma, United States), week 2; 30 mg/kg alizarin red, (Sigma, United States), week 4; 20 mg/kg calcein, (Sigma, United States), week 6. The defected calvarias were collected at week 12 and dehydrated by gradient alcohol. The undecalcified specimens were embedded in poly-methyl-methacrylate and sectioned to 150 μm thick in the orientation of the sagittal surface. Then fluorescent signals were observed using a confocal microscope (Leica, Germany).

#### Histological Analysis

The craniums from afformentioned rat model were sectioned coronally through the central area of the defect at a thickness of 5 μm with a microtome (Leica, Hamburg, Germany). Next, Masson staining, Safranine solid green staining and HE staining were performed to evaluate new bone formation according to the standard procedures.

### Statistical Analysis

SPSS 22.0 was used to analyse the data, which were expressed as the mean ± standard deviation (SD). Comparisons between different groups were assessed with one-way analysis of variance (ANOVA). Fisher’s LSD test was used for each comparison. A value of *p* < 0.05 was considered statistically significant. Detailed statistical data are reported in Supplementary Material.

## Results

### Overexpression and Quantitative Validation of SAA in Patients With ONFH

**FIGURE 1:**
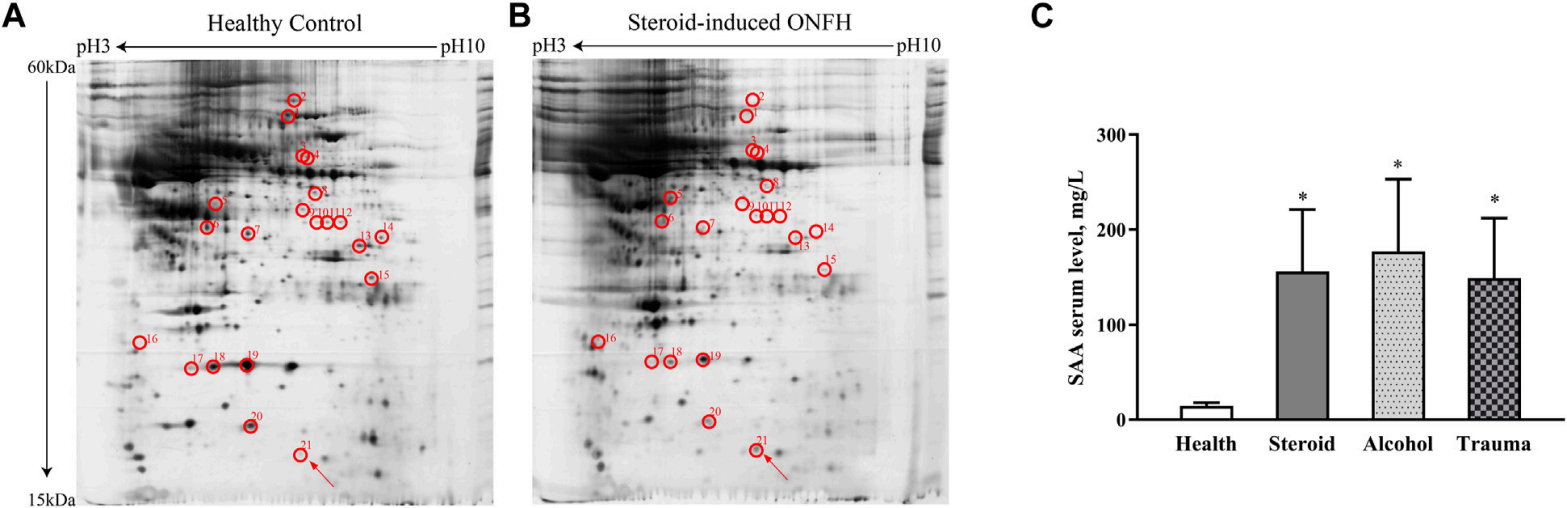
A pair of representative two-dimensional gel electrophoresis gels from the sera of 11 healthy controls **(A)** and 11 patients with steroid-induced ONFH **(B)**. The circled spots that showed identified, significant and consistent differences between two groups. SAA protein was pointed out by arrows **(C)** SAA serum level in patients with steroid-, alcohol-, and trauma-ONFH and healthy volunteers, *p < 0.05 compared with healthy volunteers.

Protein profiles from the sera of 11 patients with steroid-induced ONFH and 11 healthy volunteers were analysed with two-dimensional gel electrophoresis and silver staining. We observed 21 prominent protein spots that differed between the two groups. Three of these proteins were upregulated and the rest 18 proteins were downregulated in patients with steroid-induced ONFH (Figures 1A,B). The spots determined in the steroid-induced ONFH and health control groups were replicable. As shown in Table 2, the spot no. 21 corresponding to an upregulated protein in patients with steroid-induced ONFH was identified as SAA. Thus, SAA was identified as elevated in the serum of patients with ONFH. The serum level of SAA was quantitatively validated by ELISA and was found to be increased in the patients with steroid-, alcohol-, and trauma-induced ONFH as compared with the health controls (Figure 1C). Based on the results of the ELISA study, however, no significant difference was observed among the three ONFH groups. Based on the results of the ELISA study, the final concentration of SAA used in the following experiments was decided as 150 mg/L.

**TABLE 2:** Proteins with significant changes in the sera of patients with steroid-induced ONFH as compared with healthy controls.

Spot no	PI	MW	Protein score	Accession No	Protein name
1	6.67	86,847	424	IPI00921523	CFB Isoform 1 of Complement factor B (Fragment)
2	6.41	109,332.8	71	IPI00879709	C6 Complement component C6 precursor
4	6.55	52,384.6	87	IPI00022488	HPX Hemopexin
5	8.48	31,673	582	IPI00431645	HPR 31 kDa protein
6	6.13	38,940.5	693	IPI00478493	HPR haptoglobin isoform 2 preproproteins
7	6.13	38,940.5	439	IPI00478493	HPR haptoglobin isoform 2 preproproteins
8	6.32	41,816.7	164	IPI00479708	IGHM Full-length Cdna clone CS0DD006YL02 of Neuroblastoma of Homo sapiens
9	7.38	38,766.4	140	IPI00011264	CFHR1 Complement factor H-related protein 1
10	7.38	38,766.4	232	IPI00011264	CFHR1 Complement factor H-related protein 1
11	7.38	38,766.4	326	IPI00011264	CFHR1 Complement factor H-related protein 1
13	6.89	194,170.1	78	IPI00418163	C4B complement C4-B preproprotein
14	5.64	71,474.7	404	IPI00019568	F2 Prothrombin (Fragment)
15	4.86	78,174.4	242	IPI00017696	C1S Complement C1s subcomponent
17	6.13	45,860.8	237	IPI00641737	HPR Haptoglobin
18	6.13	45,860.8	268	IPI00641737	HPR Haptoglobin
19	6.13	45,860.8	227	IPI00641737	HPR Haptoglobin
20	6.13	38,940.5	467	IPI00478493	HPR haptoglobin isoform 2 preproproteins
21	6.28	13,580.5	292	IPI00552578	serum amyloid A protein 1 and 2

### SAA Inhibits the Osteogenic Differentiation of rBMSCs *in vitro*

**FIGURE 2:**
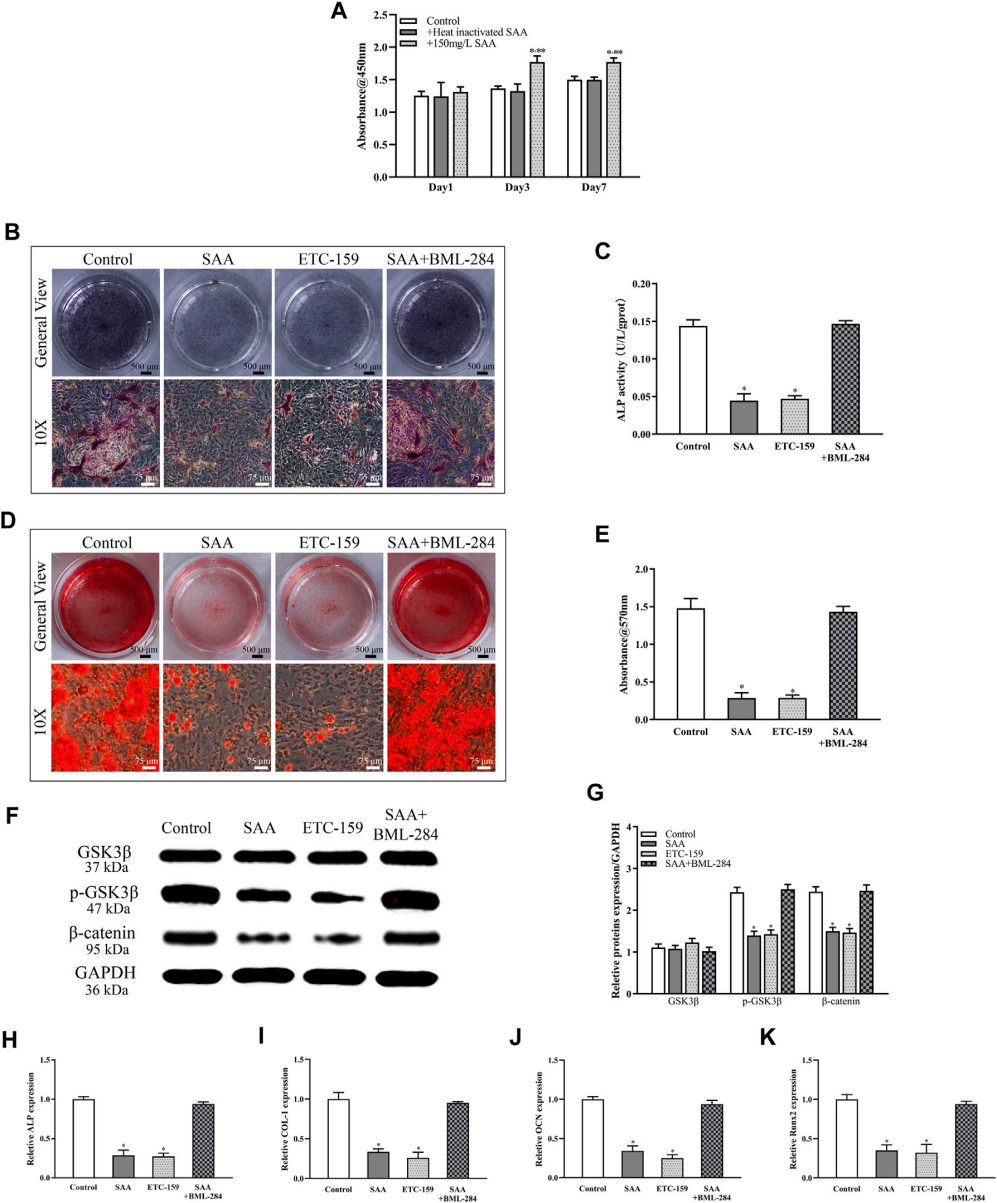
**(A)** CCK-8 **(B)** NBT/BCIP staining **(C)** ALP activity assays **(D)** Alizarin red staining **(E)** Calcium deposition was determined by measuring optical density **(F)** The stable cell lines were subjected to western blot analysis for the detection of the expression of GSK3β, *p*-GSK3β, and *ß*-catenin following normalization to GAPDH level **(G)** The intensity of relative proteins. The mRNA expression of **(H)** ALP **(I)** COL-1 **(J)** OCN(K) Runx2. *p < 0.05 compared with control group, **p < 0.05 compared with group added with heat-inactivated SAA. All experiments were performed in triplicates.

The proliferation and differentiation of BMSC plays an important role in the pathophysiological process of ONFH. Thus, to examine the effect of SAA on the proliferative rate of rBMSCs, CCK-8 assay was performed at day 1, 3, and 7. The comparison of the absorbance at 450 nm wavelength revealed the ability of 150 mg/L SAA to induce rBMSCs proliferation. After incubation with SAA, rBMSCs showed higher proliferation than those from control group and the proliferative rate of the rBMSCs exposed to 150 mg/L SAA was obviously enhanced at 7 days (Figure 2A). BCIP/NBT staining was performed to evaluate the effect of SAA on osteogenic differentiation of rBMSCs. As shown in Figures 2B,C, the bluish colouration of rBMSCs cultured with SAA was sparse as compared with the cells cultured without SAA. In agreement with the result of BCIP/NBT staining, rBMSCs treated with SAA revealed minor mineralisation than that observed with rBMSCs cultured without SAA, suggestive of the ability of SAA to suppress osteogenic differentiation (Figures 2D,E).

The mechanism underlying the inhibitory effect of SAA was further excavated. ETC-159 was added as inhibitor of the Wnt signalling pathway and BML-284 was added as activator of Wnt signalling pathway. According to Figures 2B–E, there is little difference in the inhibition of osteogenic differentiation between rBMSCs treated with SAA and ETC-159. However, when BML-284 was added as antagonist of SAA’s osteogenic inhibition, the osteogenic differentiation of rBMSCs returned to normal. Further evidence was provided by Western blot analysis, on the basis of Figures 2F,G, a decrease in the phosphorylation level of GSK3*β* was founded and downregulated the expression of *ß*-catenin owing to the addition of SAA, consistent with the results of rBMSCs added with ETC-159. While rBMSCs were co-cultured with SAA and BML-284, the protein expression of Wnt signalling pathway was not significantly different from that of control group. Real-time RT-PCR was performed to monitor the expression of osteogenesis-related genes. The expression of *ALP*, *Runx2*, *OCN*, and *COL-1* was downregulated among the groups treated with SAA than control group. Furthermore, no difference was found between group added with SAA and ETC-159, or between group added with SAA + BML-284 and control group (Figures 2H–K).

### SAA Promotes the Adipogenic Differentiation of rBMSCs *in vitro*

**FIGURE 3:**
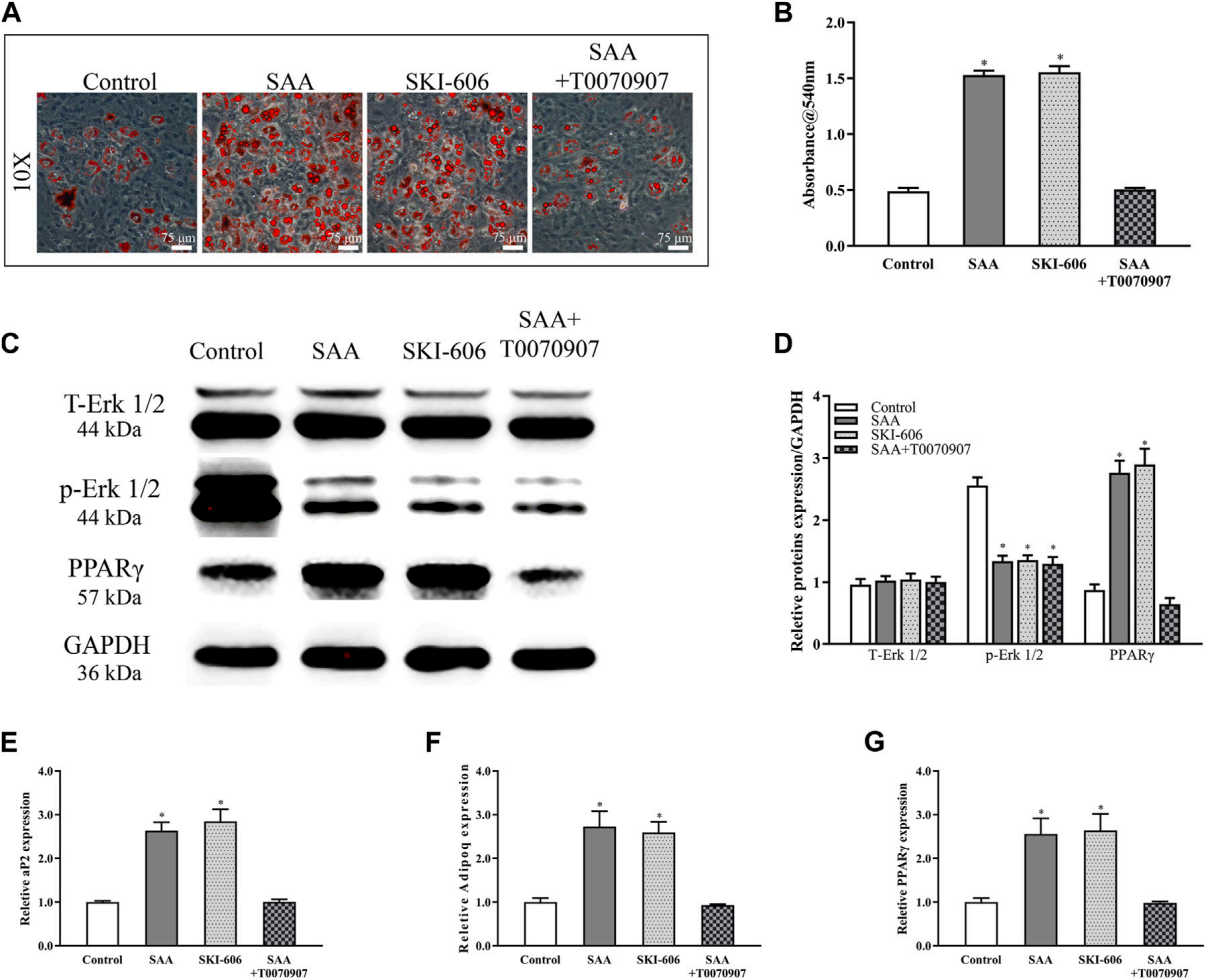
**(A)** Oil red O staining **(B)** Lipid droplet was determined by measuring optical density **(C)** The stable cell lines were subjected to western blot analysis for the detection of the expression of T-ERK1/2, *p*-ERK1/2, and PPARγ after normalization to GAPDH expression **(D)** The intensity of relative proteins. The mRNA expression of **(D)** aP2 **(E)** Adipoq and **(F)** PPARγ. *p < 0.05 compared with control group. All experiments were performed in triplicates.

Because adipocyte is found proliferated in ONFH, we further investigated whether SAA had other effects on the differentiation of rBMSCs. Oil red O staining was used to detect the effect of SAA on adipogenic differentiation of rBMSCs. As the results showed in Figures 3A,B, the number of red stained lipid droplets was significantly higher in the rBMSCs treated with SAA than in those from control group. To further test if MAPK/PPARγ pathway is affected by SAA, bosutinib (SKI-606) was used as the inhibitor of Erk phosphorylation and T0070907 as a potent selective PPARγ inhibitor was used to antagonize the adipogenic effect of SAA on rBMSCs. In accordance with Figures 3A,B SAA and SKI-606 had the same ability to promote adipogenic differentiation of rBMSCs. However, the ability was antagonized by T0070907.

Erk1/2, one of the mitogen-activated protein kinase (MAPK) family member, is known to play an important role in adipogenesis. As shown in Figures 3C,D the phosphorylation level of ERK1/2 reduced following treatment with SAA or SKI-606 or SAA + T0070907. However, the expression of downstream PPARγ was not up-regulated by SAA due to T0070907. The expression of *aP2*, *PPARγ*, and *Adipoq* was analysed in rBMSCs subjected to adipogenic induction and SAA treatment with real-time RT-PCR. As shown in Figures 3E–G, the expression of these biomarkers was significantly upregulated following stimulation with SAA or SKI-606, which attributed to the inhibitory of *p*-Erk1/2. Nevertheless, T0070907 antagonized SAA’s up-regulation of PPARγ downstream genes’ expression by inhibiting PPARγ expression.

### SAA Reduces New Bone Formation *in vivo*

**FIGURE 4:**
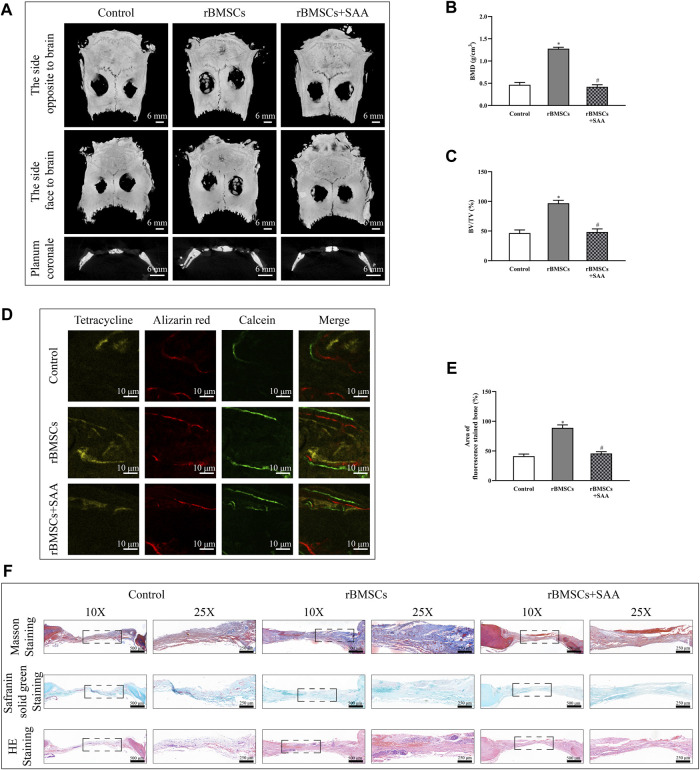
**(A)** MicroCT 3D reconstruction and coronal images of the defect area at 8 weeks after operation in each group. MicroCT analyses of bone mineral density (BMD) **(B)** and bone volume/total volume (BV/TV) **(C**,**D)** Representative image of fluorescence assay for tetracycline, alizarin red and calcein **(E)** Statistical evaluation of fluorochrome area **(F)** Histological evaluation of the defect area by Masson, Safranin solid green and HE staining. *p < 0.05 compared with control group. #*p* < 0.05 compared with the rBMSCs group. All experiments were performed in triplicates.

Lastly, the effect of SAA on bone metabolism was tested by in a bilateral critical-sized calvarial defect rat model. In short, the pre-treated rBMSCs was implanted into the critical sized, circular bone defects by using ShakeGel™ 3D hydrogel as scaffold to detect their osteogenic ability *in vivo*. 12 wk after the operation, the new bone formation in the defect area of the craniums was assessed by MicroCT scanning. As Figure 4A shown, the 3D reconstruction and coronal images rarely exhibited newly formed bone in the control group. Contrarily, the rBMSCs group showed an increase in bone formation. Besides, the rBMSCs treated with SAA group showed little evidence of new bone formation, which was almost indistinguishable from the control group. The trend was quantitively verified by BMD (Figure 4B) and BV/TV (Figure 4C) increased. The fluorescence signalling of tetracycline, alizarin red and calcein indicated the loci of new bone formation and mineralization. The formation of new bone in different time periods can be detected by fluorescence labelling injected at different time points. In accordance with Figures 4D,E, the fluorescence intensity of rBMSCs group was significantly stronger than the control group or rBMSCs + SAA group. Consistent with the above findings, the histological results of Masson, Safranin solid green and HE staining (Figure 4F) indicated that rBMSCs group showed increased mineralized bone tissues within the defect areas. However, after treating with SAA, rBMSCs lost their ability to promote new bone formation, which totally pointed that SAA reduced new bone formation *in vivo*.

## Discussion

Tracing the history of ONFH, research of its pathogenesis has been ongoing since the disease was described by Jean Cruveilhier, a French anatomist and pathologist but keeps incompletely understood (Dubois and Cozen, 1960). However, there is one agreement that local ischemia due to compromised blood flow is the final common pathway in the pathogenesis of ONFH (Hines et al., 2021). Most well-known risk factors and associated conditions of ONFH eventually leads to a kind of intra-osseous compartment syndrome inside the femoral head due to an ischemic cascade: *1*) overgrowth of marrow adipocyte *2*) intra-osseous hypertension; *3*) vascular compression and intravascular hypercoagulability; *4*) bone marrow necrosis and osteocyte death and *5*) fibrovascular reparation around the necrotic zone (Yoon et al., 2020). Therefore, finding the factors that cause the affected differentiation of BMSCs is potential key to further research.

Proteomics analysis, in which total protein in tissue are identified and quantified directly, has shown to be a valuable way to elucidate the molecular basis of disease etiology and pathogenesis. ONFH (Chen et al., 2015a). Previous studies have focused on the change of protein in bone tissue and cartilage (Zeng et al., 2019; Song et al., 2021). Admittedly, there have been many proteomic studies on various pathological tissues of ONFH. In previous studies, tissue sources have tended to be local bone or cartilage rather than serum samples taken from peripheral blood. However, the risk factors of ONFH, excluding trauma, tend to be systemic rather than local. Hence, the proteomic research to investigate the expression of mutated proteins in the sera of patients with ONFH is indispensable. In the present study, proteomic analysis was performed to evaluate the systemic changes in the expression levels of proteins. To improve the reproducibility, sensitivity, and objectivity of the research, the sample selection criteria were strictly followed, and the highly abundant proteins were removed to avoid masking of the proteins expressed in low levels. Accordingly, 21 proteins were found to exhibit variable expression patterns in patients with steroid-induced ONFH as compared with healthy volunteers. Although 18 of these proteins were identified, we focused specifically on SSA, which showed upregulated expression in the serum of patients with steroid-induced ONFH as compared with normal subjects.

SAA is present in the blood of healthy individuals at low levels (20–50 mg/L), but its expression increases to about 1,000-fold within 24 h from the onset of acute phase response (APR), which includes a series of physiologic changes as a consequence of infection, inflammation, trauma, or other events (Gabay and Kushner, 1999; Yoo and Desiderio, 2003; Kushner, 1982). There are two possible explanations for the phenomenon we found above. Firstly, there is mutual similarity between the risk factors of ONFH and the factors leading to the increase of SAA. No matter alcohol, hormone or trauma can cause the increase of SAA expression (Knesek et al., 2010; Kim et al., 2014; Li et al., 2021), which was verified by ELISA. The causes may be related to liver damage caused by heavy drinking, abnormal lipid metabolism caused by alcohol and corticosteroids, and APR caused by acute trauma. Secondly, the necrosis of bone marrow and osteocytes, absorption of necrotic area, and synovial inflammation may cause focal inflammation occurred during ONFH resulted in the upregulation of SAA expression (Chen et al., 2015b; O'Hara et al., 2010).

Adipogenesis and osteogenesis of BMSCs were shown as significant factors affecting the process of osteonecrosis. The inhibition of osteogenic differentiation followed by increased lipid generation resulted in a decrease in bone formation. Moreover, blood circulation in femoral head was impaired following accumulation of the fatty tissue, eventually resulting in ONFH (Cui et al., 2006). The influence of SAA on bone metabolism was investigated here to explore its role in maintaining the balance between osteogenesis and adipogenesis of BMSCs. SAA exhibited the capability of promoting proliferation, curbing osteogenesis, and facilitating adipogenesis.

The Wnt/*β*-catenin signalling pathway is considered as a mediator of osteogenic differentiation (Chen et al., 2012; Luo et al., 2015). The decrease in *ß*-catenin expression, which is in line with GSK3*β* phosphorylation, results in the inhibition of ALP activity and mineralisation (Ni et al., 2010). The inhibitory effect of SAA on osteogenesis was initially assessed using BCIP/NBT and alizarin red staining. The molecular mechanism underlying the inhibitory effect of SAA was investigated; the addition of SAA resulted in the downregulation of the phosphorylation of GSK3*β*, which repressed the expression of *ß*-catenin in the downstream signalling pathway. The expression of *ALP*, *Runx2*, *OCN*, and *COL-1*, the biomarkers of osteogenic differentiation (Lee et al., 2009; Heo et al., 2010; Satija et al., 2013), was simultaneously downregulated following treatment with SAA proteins, as observed with PCR results. For adipogenic differentiation, the conversion of BMSCs to preadipocytes that differentiate into adipocytes is triggered by the enforced expression of PPARγ, which is regulated by the MAPK/PPARγ signalling pathways (Kang et al., 2007; Gierloff et al., 2014). Scilicet, the decreased phosphorylation Erk1/2 upregulated the expression of PPARγ and facilitated the adipogenic differentiation, as demonstrated in the present study. The expression of adipogenesis-related genes, not only *PPARγ* but also *aP2* and *Adipoq* (Lee et al., 2009; Muruganandan et al., 2009; Tokuzawa et al., 2010), was detected and confirmed the positive effects of SAA on the adipogenic differentiation of rBMSCs. The two pathways worked together to break the balance of bone metabolism of which the effect was reflected in a markable reduction of bone formation *in vivo*.

**FIGURE 5:**
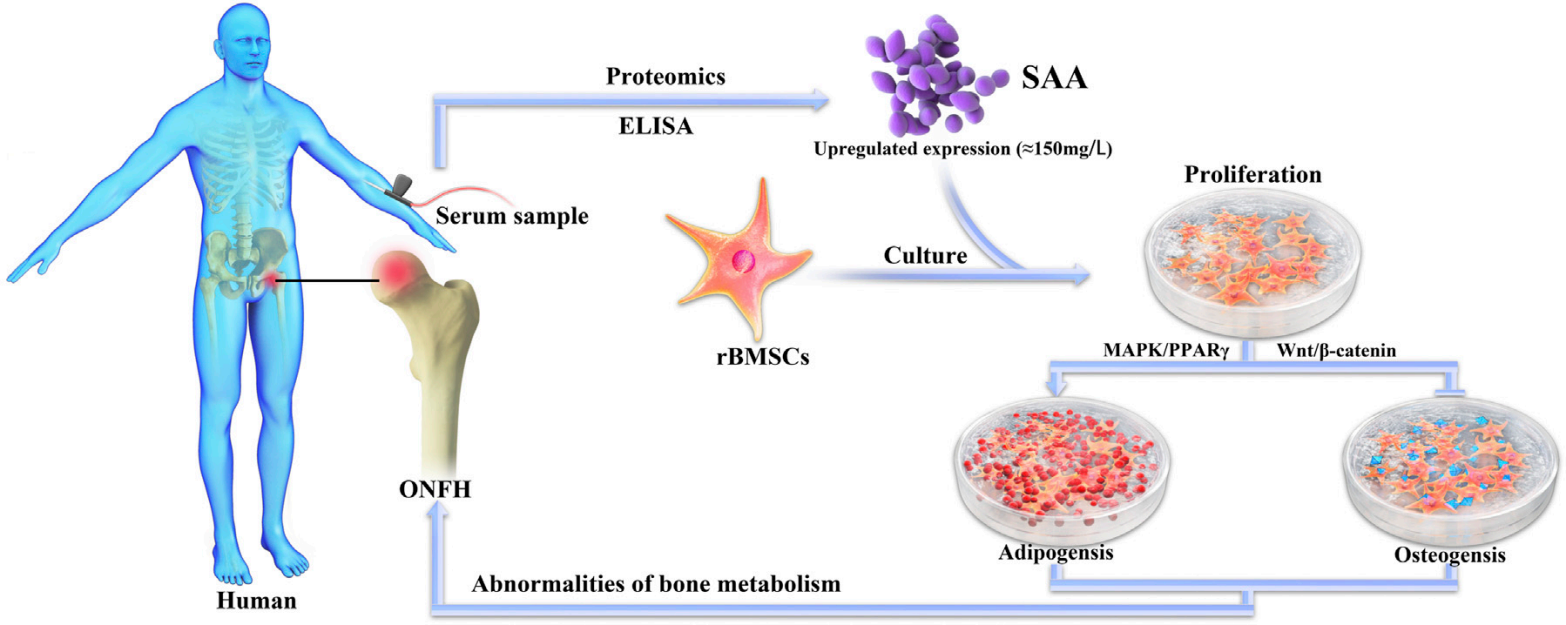
A graphical summary of the whole study.

In this study, we first started from serum samples of ONFH patients and verified by proteomics and ELISA experiments that SAA was significantly increased in the serum of ONFH patients. Then, SAA was found to inhibit osteogenic differentiation and promote adipogenic differentiation of BMSCs *in vitro*. Further studies on the molecular mechanism of this phenomenon by Western Blot and PCR showed that SAA could inhibit Wnt/*ß*-catenin signalling pathway and activate downstream PPARγ of MAPK signalling pathway. Finally, we verified *in vivo* that SAA can cause abnormal bone metabolism of BMSC and reduce bone formation (Figure 5). Hence, SAA is a vital protein in the physiological process of ONFH and can act as a potential therapeutic target to treat ONFH.

## Data Availability

The datasets presented in this study can be found in online repositories. The names of the repository/repositories and accession number(s) can be found in the articles/Supplementary Material.
