# Supplementary material for: Serum Amyloid A Correlates With the Osteonecrosis of Femoral Head by Affecting Bone Metabolism
Source: Front Pharmacol. 2021 Oct 18;12:767243. doi: 10.3389/fphar.2021.767243 (PMC8559508; doi:10.3389/fphar.2021.767243)
Supplement: Supplementary file 1 [file DataSheet1.docx]

| **Supplementary Table 1: Clinical features and demographic information of healthy volunteers and steroid-induced ONFH patients** | |
| --- | --- |
| Clinical features | Numbers |
| Healthy volunteers |  |
| Mean age (range, year) | 34.00 ± 7.54 (21-45) |
| Gender (male/female) | 5/6 |
| Health status | Good |
| Total | 11 |
|  |  |
| Patients with steroid-induced ONFH | |
| Mean age (range, year) | 35.45 ± 8.35 (25-56) |
| Gender (male/female) | 7/4 |
| Location |  |
| Left | 1 (9.1%) |
| Right | 2 (18.2%) |
| Bilateral | 8 (72.7%) |
| Total | 11 |

| **Supplementary Table 2: Clinical features and demographic information of steroid-induced ONFH patients, alcohol-induced ONFH patients, trauma-induced ONFH and healthy volunteers.** | |
| --- | --- |
| Clinical features | Numbers |
| Healthy volunteers |  |
| Mean age (range, years) | 34.95±8.86 (21-54) |
| Gender (male/female) | 10/10 |
| Health status | Good |
| Total | 20 |
|  |  |
| Steroid-induced ONFH patients |  |
| Mean age (range, years) | 36.20±8.56 (22-55) |
| Gender (male/female) | 13/7 |
| Location |  |
| Left | 2 (10%) |
| Right | 1 (5%) |
| Bilateral | 17 (85%) |
| Total | 20 |
|  |  |
| Alcohol-induced ONFH patients |  |
| Mean age (range, years) | 39.70±8.45 (26-55) |
| Gender (male/female) | 20/0 |
| Location |  |
| Left | 1 (5%) |
| Right | 3 (15%) |
| Bilateral | 16 (80%) |
| Total | 20 |
|  |  |
| Trauma-induced ONFH patients |  |
| Mean age (range, years) | 21.35±10.73 (12-54) |
| Gender (male/female) | 15/5 |
| Location |  |
| Left | 11 (55%) |
| Right | 9 (45%) |
| Bilateral | 0 (0%) |
| Total | 20 |

**Flow cytometry identification**

For phenotypic characterization, 2×10^5^ rBMSCs were cultured with fluorescein CD34 (Bioscience Pharmingen), CD44 (eBioscience, USA) and CD90 (eBioscience) at a dilution rate of 1: 100 for 30 min at 4°C. Then, flow cytometry was performed using FACSCalibur flow cytometer (Becton Dickinson, USA). Flowjo 7.6.5 software (Tree Star Inc., Ashland, OR, USA) was used for data analysis. The cells were negative for CD34 but positive for CD44 and CD90, which are typical biomarkers for rBMSCs.


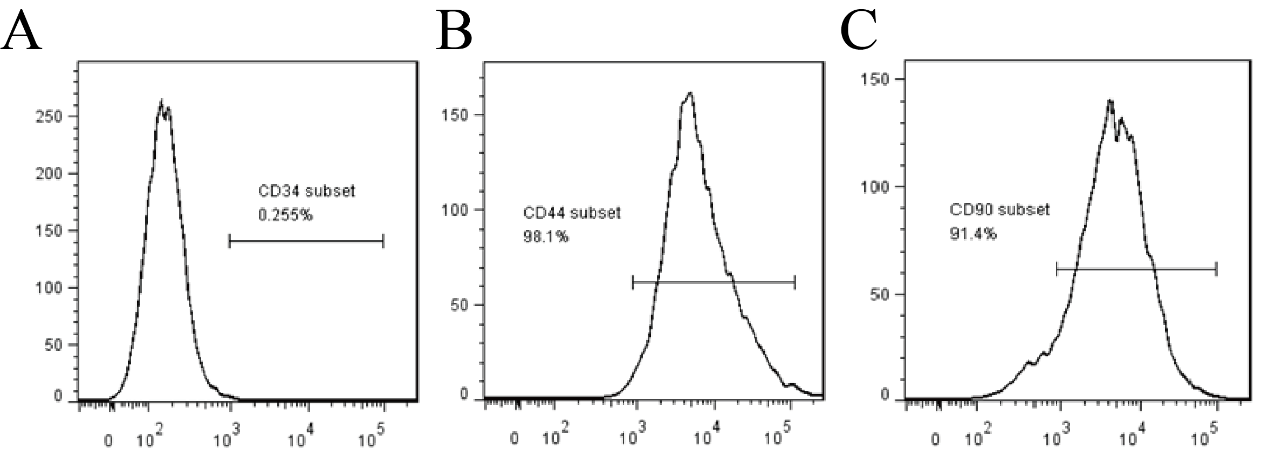


**Supplementary Figure 1:** Characteristics of isolated rBMSCs. The isolated rBMSCs were negative for CD34(A) but positive for CD44(B) and CD90(C). The X axis represents fluorescence intensity.
